# Surgical solution for retrograde peri-implantitis: A case report on managing implant-associated apical infection

**DOI:** 10.12669/pjms.42.(11AASC).15675

**Published:** 2026-04

**Authors:** Hassan Yaqoob, Syed Murtaza Raza Kazmi, Shahrukh Ali Khan, Ali Sadiq

**Affiliations:** 1 Hassan Yaqoob, BDS Resident, Prosthodontics, Department of Dentistry and Oral Health Sciences, The Aga Khan University, Karachi, Pakistan; 2 Syed Murtaza Raza Kazmi, BDS, FCPS, MHPE, FICOI, FICCDE, FHEA, FDTFed, Associate Professor and Consultant Prosthodontics Department of Dentistry and Oral Health Sciences, The Aga Khan University, Karachi, Pakistan; 3 Shahrukh Ali Khan, BDS Section of Prosthodontics, Fatima Jinnah Dental College, Karachi, Pakistan; 4 Dr. Ali Sadiq Director, Associate of Science in Dental Hygiene (ASDH) Assistant Professor Periodontology Dental Section, Department of Surgery, The Aga Khan University, Karachi, Pakistan

**Keywords:** Apical peri-implantitis, Guided bone regeneration, Implant complications, Periapical lesion, Retrograde periimplantitis, Surgical debridement

## Abstract

Retrograde peri-implantitis (RPI) represents an infrequent yet clinically consequential postoperative complication characterized by apical pathology around an implant exhibiting otherwise intact coronal osseointegration. This case report details the diagnosis and management of active RPI in a 66-year-old female with history of hypertension and diabetes mellitus, presenting with persistent postoperative pain, swelling, and sinus tract formation one month after delayed implant placement in a site previously affected by apical periodontitis. Radiographic and CBCT evaluation revealed a well-defined periapical radiolucency with preservation of marginal bone levels. Initial conservative therapy resulted in transient symptom relief, with recurrence following prosthetic rehabilitation. Definitive management involved surgical access, thorough mechanical debridement, chemical surface detoxification, and guided bone regeneration using combined autogenous and allogeneic grafting beneath a resorbable membrane. The intervention yielded uneventful healing, progressive radiographic bone regeneration, and complete lesion, without compromising implant stability. This case highlights the critical importance of early recognition, comprehensive diagnostic assessment, and timely regenerative surgical intervention for achieving predictable outcomes in RPI, particularly in sites with prior periapical pathology.

## INTRODUCTION

Dental implants have demonstrated remarkable efficacy over the years in restoring missing teeth in both completely and partially edentulous patients. Despite their high success and survival rates, emerging evidence has drawn attention to multiple localized complications, including retrograde peri-implantitis (RPI), a rare but clinically significant apical infection now recognized as an emerging concern in implant dentistry.^1^

Retrograde peri-implantitis, first described by McAllister in 1992, is characterized by a periapical radiolucency that develops shortly after implant placement, while the coronal portion of the implant demonstrates normal osseointegration.^2^ Clinically, RPI presents with radiographic evidence of apical bone loss accompanied by symptoms such as pain, swelling, tenderness, erythema, or fistula formation. In certain cases, the infection remains inactive, exhibiting apical radiolucency without overt clinical manifestations. Management strategies for RPI range from observation and conservative therapy to surgical intervention, depending on the severity of the lesion. Despite its clinical relevance, RPI remains underreported, particularly in developing regions, likely due to limited awareness and diagnostic challenges among clinicians.^2^

The present report describes a rare case of RPI identified during the osseointegration phase of implant healing, explores potential etiological factors, and evaluates the clinical outcome following surgical management aimed at complete resolution of the lesion.

## CASE PRESENTATION

A 66 years old female patient presented to the dental clinics of our tertiary care hospital with the chief complaint of difficulty in mastication on both sides. Her medical history was significant for diabetes mellitus, hypertension, and depression, for which she was on regular medication. Intraoral examination revealed compromised oral hygiene, characterized by the presence of plaque and both supra- and subgingival calculus deposits. Additionally, a porcelain-fused-to-metal (PFM) bridge was observed on the left mandibular arch, connecting a natural tooth to an implant. The abutment tooth (#35) was previously root-canal treated and exhibited secondary caries adjacent to the restoration, with periapical radiolucency suggestive of underlying periapical pathology.

Considering the unfavorable biomechanics of the tooth implant supported prosthesis and the poor prognosis of the compromised abutment, replacement of the existing restoration was planned following extraction of the abutment tooth. Although immediate implant placement was initially considered, the presence of a buccal dehiscence at the extraction site warranted a delayed approach with meticulous debridement and socket preservation. Following adequate bone maturation over a period of three months, a 4.1 x 11.5 mm implant (Zimmer Biomet) was placed with a torque of 40 N-m. The surgical procedure was performed in a controlled clinical setting under strict aseptic conditions, including sterile draping, use of sterile surgical instruments, and adherence to standard infection control protocols. A healing abutment was then connected, and the surgical site was closed using vicryl sutures. The initial postoperative phase was uneventful, showing satisfactory healing without infection; however, one month after fixture placement, the patient reported persistent pain and localized swelling at the surgical site. Localized non-surgical periodontal debridement was performed around the implant region, and the patient was kept under follow-up for subsequent evaluation.

**Fig.1:**
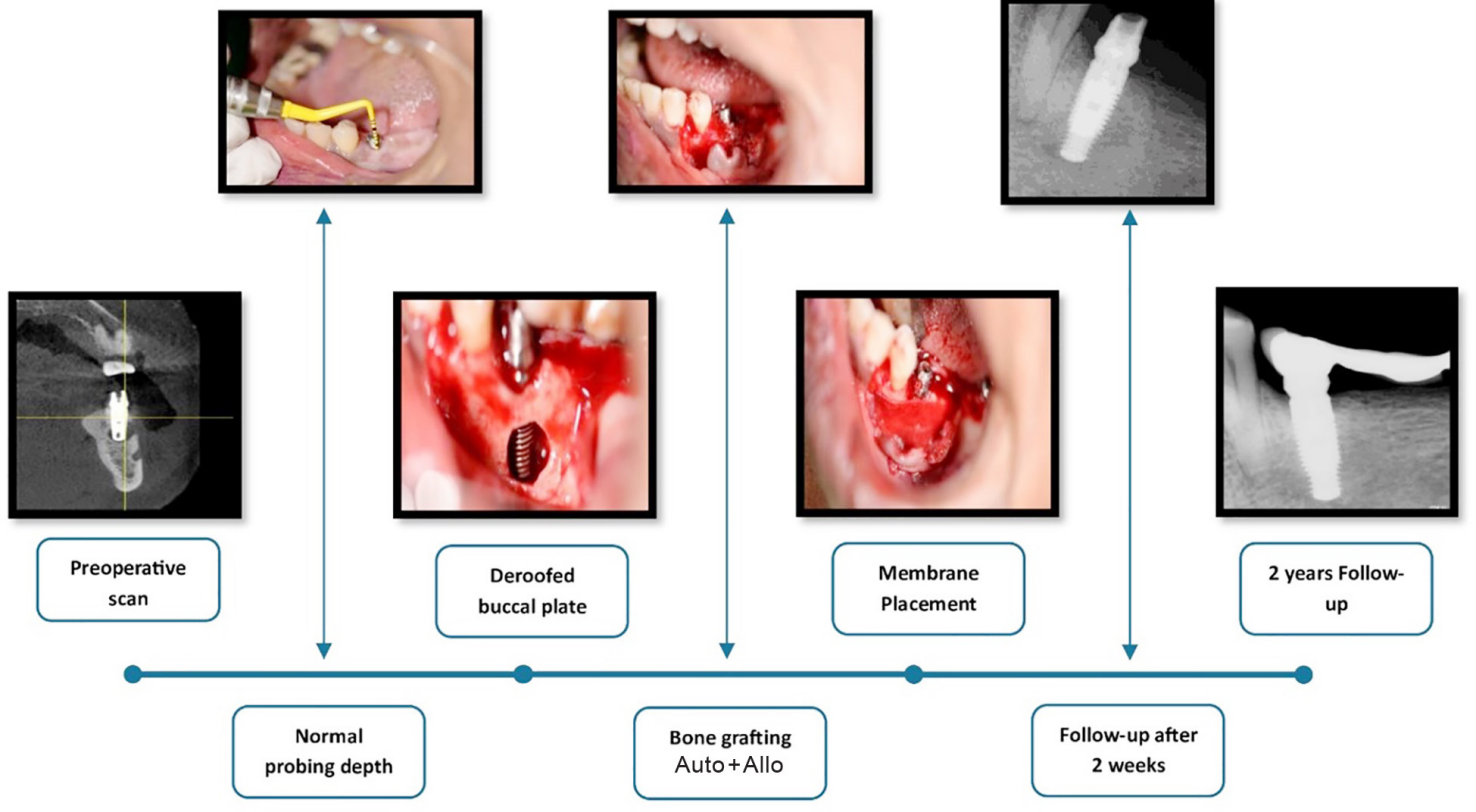
Clinical and radiographic sequence demonstrating the management of retrograde peri-implantitis. The lesion was accessed via buccal plate deroofing, followed by meticulous debridement, bone grafting (autograft + Allograft), and membrane placement. Postoperative radiographs at 2 weeks and 4 months show progressive bone fill and complete bone remodeling with implant stability at 2 years.

Despite initial management, the patient’s condition deteriorated, with worsening pain and localized swelling in the left mandibular 35 region at the implant apex, presenting as a fluctuant abscess with associated sinus tract formation, consistent with an active periapical infection. A five-day course of systemic antibiotic therapy with amoxicillin (500 mg) and metronidazole (500 mg), administered three times daily, resulted in only transient symptomatic improvement. Following apparent resolution, the patient was rehabilitated with a three-unit implant retained fixed dental prosthesis in the left mandibular posterior region, replacing the 35–37 region. However, the symptoms recurred, as localized tenderness and a fluctuant abscess at the implant apex. Notably, the patient’s poorly controlled diabetes at the time contributed to the persistence of infection and impaired healing response. Given the recurrent lesion and persistent abscess, definitive surgical debridement with local disinfection was planned to ensure long-term resolution and implant stability (Fig.1).

After obtaining informed consent, a full thickness mucoperiosteal flap was reflected with releasing incision, extending from the left mandibular canine to the distal aspect of the implant site. Intraoperatively, the periapical lesion was found to have perforated the buccal cortical plate of the mandible. The defect was deroofed, followed by meticulous mechanical debridement and degranulation using a Universal Implant Deplaquer (Straumann, USA). Chemical detoxification of the implant surface was achieved by applying 0.2% chlorhexidine gluconate for two minutes, followed by copious irrigation with sterile 0.9% saline solution. Upon complete decontamination, the osseous defect was grafted using a combination of harvested autogenous bone and allograft material (Rocky Mountain Bone Graft, USA). A resorbable membrane was placed to cover the graft, and primary closure was achieved with 5-0 Prolene sutures. Postoperative care included systemic antibiotic therapy and chlorhexidine mouth rinses.

At the postoperative review, the patient reported marked symptomatic improvement with no recurrence of abscess. The implant remained stable. Radiographs obtained intraoperatively and at one- and four-month intervals revealed progressive healing with early reparative tissue formation around the implant apex. Complete apical bone remodeling was observed at the two-year follow-up (Fig.1).

## DISCUSSION

The current case report describes a rare case of RPI, a condition that remains infrequently documented in the literature. It represents distinct pathological entity among implant-related infections, characterized by periapical inflammation of an otherwise Osseo-integrated implant. Reported prevalence rates are low, ranging from 0.34% to 0.9%, reflecting both the rarity of the condition and potential underdiagnosis in clinical practice.^3^,^4^ It typically develops during the early post-placement phase, where localized apical inflammation, if left untreated, may lead to implant mobility and lack of osseointegration.^3^

The etiology of RPI is complex and considered to be influenced by multiple contributing factors. One of the most frequently implicated causes is the persistence of residual infection or microorganisms within the extraction socket or from a previously infected tooth, which may serve as a nidus for reinfection during osseointegration.^5^ Contamination of the implant surface by microorganisms originating from periapical lesions of adjacent teeth has also been documented. Moreover, thermal injury or localized bone necrosis resulting from excessive drilling torque or inadequate irrigation during osteotomy preparation has been associated with apical bone inflammation, thereby predisposing the site to subsequent bacterial colonization.^6^

In the present case, an atraumatic extraction was performed, followed by meticulous curettage of the alveolar socket to minimize the residual microbial load. The adjacent teeth were vital and radiographically intact; therefore, prophylactic endodontic treatment was not indicated. The implant, however, was placed in a site previously affected by apical periodontitis, which likely served as the origin of infection. This finding is consistent with the report by Lefever et al., who identified a significantly increased risk of RPI, with an odds ratio ranging from 7.2 to 8, in cases involving endodontic pathology of the extracted or neighboring tooth.^7^ The pre-existing periapical lesion in this case may have facilitated bacterial persistence within the bone despite thorough debridement. Reactivation of these dormant microorganisms during osteotomy preparation likely led to microbial colonization of the implant apex and the subsequent development of RPI.

Diagnosis is established through a combination of clinical and radiographic assessment. Clinically, RPI may manifest as swelling, pain, erythema, suppuration, or sinus tract formation, whereas in certain cases, it remains asymptomatic and is detected radiographically as a well- or ill-defined periapical radiolucency.^3^ Determining whether the lesion represents an active or inactive stage is essential for guiding appropriate management. In the present case, the diagnosis corresponded to the active stage of RPI, characterized by persistent pain, swelling, and the presence of a sinus tract, accompanied by a distinct periapical radiolucency. The diagnosis was confirmed by correlating clinical signs with radiographic findings. Cone-beam computed tomography (CBCT) further delineated the extent of the apical lesion and confirmed the absence of coronal bone loss, thereby distinguishing RPI from conventional peri-implantitis.

Current literature describes a variety of treatment options for RPI, ranging from conservative therapy to surgical intervention. Although multiple protocols have been proposed, no standardized criteria for definitive management exist.^8^ Treatment is generally guided by clinical symptoms, radiographic findings, and implant stability. Conservative measures such as antibiotics and observation may suffice for small, asymptomatic lesions^5^, whereas surgical intervention is indicated for active or progressive cases. Surgical procedures typically include mechanical debridement and surface decontamination, often supplemented with bone grafting to restore apical bone integrity.^9^ In more advanced situations, implantoplasty, apicoectomy, or implant removal may be required to achieve complete resolution.^8^,^10^

In our case, surgical debridement with apical curettage and implant surface decontamination proved effective due to the persistence of symptoms and the presence of a well-defined periapical lesion. As the implant demonstrated stability and the infection was localized, removal was not warranted. The combined use of autogenous and allogeneic graft materials with a resorbable membrane provided both osteogenic potential and scaffold stability, facilitating guided bone regeneration. This approach effectively eliminated residual infection, promoted apical bone healing, and preserved implant stability.

**Fig.2:**
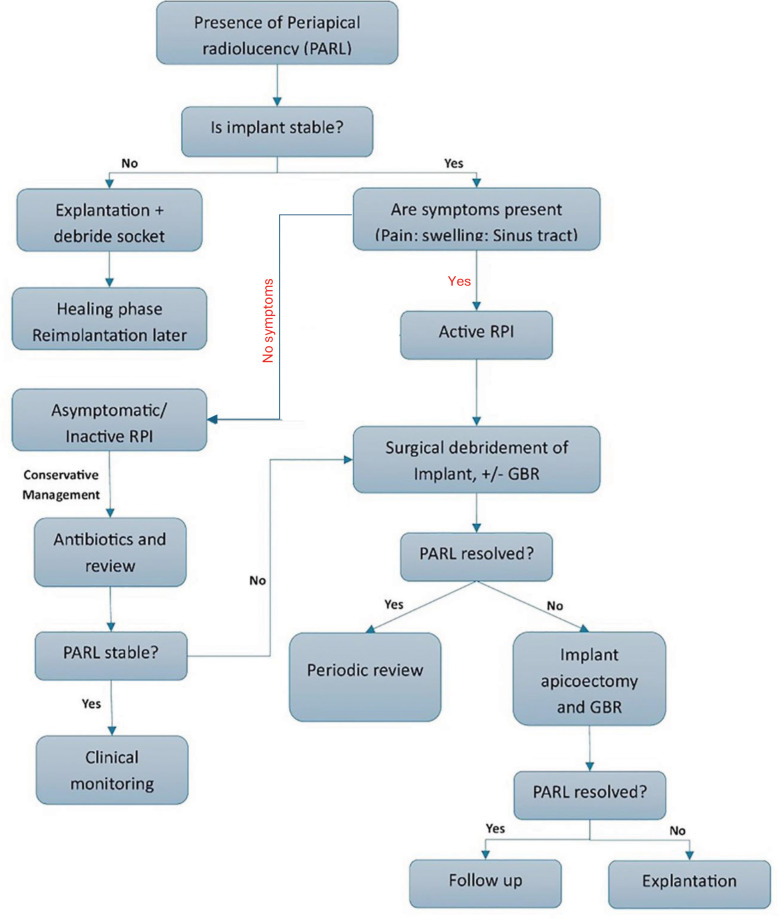
Decision tree outlining the diagnostic and management pathway for retrograde peri-implantitis (RPI). This schematic was developed by the authors based on treatment strategies and clinical recommendations reported in the existing literature.

Early diagnosis is crucial for preserving implant stability and preventing apical disease progression. Preoperative evaluation of sites with previous endodontic pathology, thorough socket debridement, delayed implant placement in infected areas, and strict aseptic protocols can greatly reduce the risk of RPI and contribute to long-term implant success. A structured decision-making approach (Fig.2) may assist clinicians in selecting appropriate interventions based on implant stability and severity of symptoms.

## CONCLUSION

Retrograde peri-implantitis remains an uncommon but clinically significant complication that can compromise implant success. This report highlights the role of comprehensive diagnostic evaluation, meticulous surgical planning, and the use of regenerative biomaterials in achieving long-term peri-implant health. Greater clinician awareness and early detection are crucial to prevent apical pathology and ensure predictable implant survival.

### Authors’ contributions:

**HY:** Writing - Original Draft, Visualization, Methodology

**SM:** Conceptualization, Writing - Review & Editing

**SA:** Resources, Methodology

**AS:** Writing - Review & Editing, Supervision and responsible for the accuracy of study.

All authors have approved the final version of manuscript.
